# Contactless crystallization method of protein by a magnetic force booster

**DOI:** 10.1038/s41598-022-21727-x

**Published:** 2022-10-14

**Authors:** Syou Maki, Masayuki Hagiwara

**Affiliations:** 1 Institute of Frontier Science and Technology, Okayama University of Science, 1-1 Ridai-cho, Kitaku, Okayama, 700-0005 Japan; 2 Center for Advance High Magnetic Field Science (AHMF), Graduate School of Science, Osaka University, 1-1 Machikaneyama-cho, Toyonaka, Osaka 560-0043 Japan

**Keywords:** Biological techniques, Engineering, Physics

## Abstract

We developed a new type of compact magnetic force booster by which we succeeded in crystallizing proteins (hen egg white lysozyme) while making them levitate in a solution without contacting the container. This technique is noteworthy in the practical merit that we could control the growth of crystals from the initial stage of nucleation in a magnetic field of merely a few Tesla. The shape of the booster was designed in accordance with the dynamical stability against external forces acting on the crystals. Under a stable condition, the crystals condensed spherically, and formed a “*shell* *shaped*” crystallization with a hollow interior. Our magnetic force booster has the potential for use in innovation, especially in the field of protein crystal engineering.

## Introduction

In the field of structural biology, a three-dimensional arrangement and its configuration of protein molecules are determined by the use of high-intensity X-ray synchrotron radiation. High-quality protein crystals are very desirable, and high-resolution diffraction pattern has brought many informative benefits to analysis in crystallography. In the creation of quality crystals, the crystal growth is expected to be conducted without any mechanical stress. Ideally speaking, protein crystals should be grown as stably levitating in a solution while being free from contact with the container wall. Such a “*contactless*” condition is also known as a “*containerless*” condition or a “*free-surface*” condition, and many scientists have been attempting to achieve that by means of ultrasonic pressure^1–5^, electrostatic levitation^6^, two-phase liquids interface^7,8^, crystallization in a phase-separated droplet^9–14^, and magnetic force^15^. By the technique of the magneto-Archimedes levitation^16^, Maki and Ataka crystallized hen egg white lysozyme (HEWL) at the solution side of an air–liquid interface^17,18^. This method achieved a partial contactless condition, and demonstrated that magnetic levitation is of utility even for the engineering of protein crystallization, although we could not reach the final goal of complete “*contactless* *crystallization*”. Around the same time, the National Institute for Materials Science (NIMS) also attempted the contactless protein crystallization by using a magnetic force booster^19^. They thought that accurate weightlessness would be a breakthrough in crystallization, and they aimed to realize a “*uniform*” magnetic force field. A “*uniform magnetic force field*” is a field in which every magnetic force has the same orientation and magnitude, and it is different from a “*uniform magnetic field*”. The NIMS’s booster had two small pieces made of a ferromagnetic substance and strong support pillars and stage, which are indispensable to hold the booster. Magnetic force near the body of the booster is enhanced owing to its large magnetization and spatial gradient. Kiyoshi et al*.*, who were the inventors of the booster used by the NIMS, realized an accurate weightless environment by applying the same magnitude of upward uniform magnetic force as the gravity^20,21^. However, contactless crystallization was virtually impossible to achieve only by employing the weightless condition of the crystal, and resulted in failure by the growth of many microcrystals on the vessel wall. The reason for that is natural in the solution; even in a zero gravity space, Marangoni convection happens, and the influence of thermal fluctuation, such as Brownian motion, is inevitable.

In the present study, we developed new types of magnetic force boosters from various experimental viewpoints. We tested many kinds of uniquely designed boosters, which were made carefully to balance the gravitational force and the levitating force on the crystal nuclei. We noticed through the experiments that “*dynamical* *stability*” on the nuclei might be a necessary condition for realizing contactless crystallization. By jointly utilizing the magnetic forces from the booster and a superconducting magnet, we succeeded in the stable crystallization of HEWL protein without contacting the container wall, applying only the magnetic fields of a few Tesla. Here we will describe the details of our contactless crystallization method by the booster and report new findings through in situ observation of the crystallization of HEWL protein.

## Theoretical ideas

### Basic equations

The vector of magnetic flux density ***B*** (T) in a superconducting magnet is calculated by the Biot-Savart equation. In this study, the ***B*** is decomposed three-dimensionally in the cylindrical coordinate system as ***B*** (*b*_*r*_, *b*_*θ*_, *b*_*z*_). The vector of the magnetic force ***F*** (*f*_*r*_*,* *f*_*θ*_*,* *f*_*z*_) is a body force, and is proportional to the product of the magnetic susceptibility χ (m^3^/kg), the magnetic flux density, and its gradient. This is presented as the following equation^22^.

1 $${\varvec{F}} = \frac{\upchi }{{\,\upmu _{0} }}\,\uprho \,\left( {{\varvec{B}} \cdot \nabla } \right)\,{\varvec{B}}.$$

**Figure 1:**
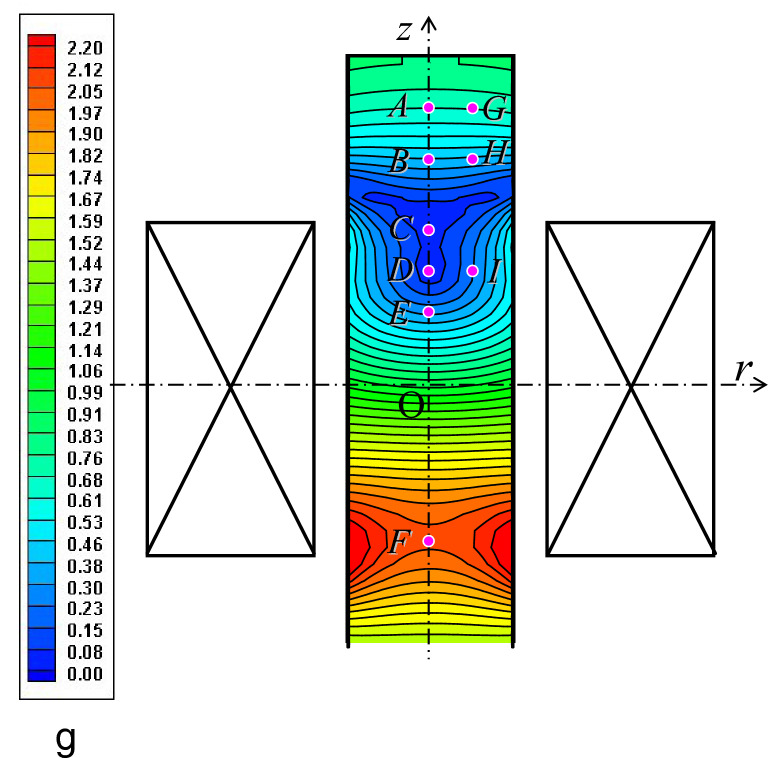
The computational isothermal distribution of the magnitude of the magnetic force. Points *A* to *I* are representative points, and *C* is the point of maximum magnetic force (MMF). The magnetic force magnitude is computed so that *f*_*z*_ becomes zero at the MMF point. The blue isothermal distribution corresponds to a weak gravity environment, and the red distribution corresponds to a hypergravity environment. The hourglass-shaped curves near *C* indicate that the *MF*_*z*_ becomes weak, whereas the *MF*_*r*_ is maintained.

Here, μ_0_, ρ, and ∇ are the magnetic permeability of vacuum 4π × 10^−7^ (H/m), the density, and a differential operator (nabla), respectively. Along the central axis of the superconducting magnet (*z*-axis in Fig. 1), the above equation is often approximated as^23^:

2 $${\varvec{F}} = \;\left( {\begin{array}{*{20}c} {f_{r} } \\ {f_{\theta } } \\ {f_{z} } \\ \end{array} } \right)^{\text{T}} = \left( {\begin{array}{*{20}c} 0 \\ 0 \\ {\frac{\upchi }{{\upmu _{0} }}\,\uprho \,b_{z} \frac{{d\,b_{z} }}{d\,z}} \\ \end{array} } \right)^{\text{T}} .$$

### Balance of force on the crystals

In a conventional superconducting magnet [see (A) in Appendix 1 attached as the Supplementary Material], there are two points nearby the coil edge on the bore axis where the vertical component of the magnetic force is at its maximum. We shall name such points the “maximum magnetic force point” (MMF point) in this paper. The MMF point can be obtained by the Biot–Savart equation theoretically, but in this study, we determined the point based on the measured data provided by the magnet company Japan Superconductor Technology Inc (JASTEC). In the schema of the superconducting magnet in Fig. 1, the positions *C* and *F* correspond to the MMF points, which are symmetrically positioned with respect to the magnet coil center, which is the origin, O. We utilized the magneto-Archimedes effect in our crystallization process^16^. Concretely, gadolinium chloride, which is a paramagnetic substance with large magnetic moments, was adopted as a precipitating agent. By applying magnetic fields, the paramagnetic substance (e.g., the solution) was attracted towards the magnet center, whereas the diamagnetic substance (e.g., HEWL molecules) was repelled by a large magnetic flux density. That is, the external forces acting on the HEWL crystals were assigned into (a) a gravitational force, (b) a buoyancy force from the solution, (c) a magnetic force acting on the crystals, and (d) a repulsive force of the magnetic force acting on the solution. The crystals’ behavior was guided by the balance of the resultant forces among (a) to (d). In this study, we decomposed the forces in the vertical, circumferential, and radial directions in the cylindrical coordinate system, and the driving force vector on the crystals shall be expressed as ***RF***(*MF*_*r*_*,* *MF*_*θ*_*,* *MF*_*z*_). In this system, the circumferential component *MF*_*θ*_ becomes zero due to the axisymmetry in a solenoidal magnet. We assume the vertically upward force direction to be positive. The resultant force between (a) and (b) represents the term of buoyancy force, and contributes to the vertical direction only. This will be abbreviated as *BF*. The resultant force between (c) and (d) is the term of magnetic force, the vertical and radial components of which shall be abbreviated as *MF*_*z*_ and *MF*_*r*_, respectively. In the cylindrical coordinate system, ***RF*** is summarized as follows.

3 $${\varvec{RF}} = \left( {\begin{array}{*{20}c} {MF_{r} } \\ {MF_{\theta } } \\ {BF + MF_{z} } \\ \end{array} } \right)^{\text{T}} = \left( {\begin{array}{*{20}c} {\frac{{\left( {\,\rho_{c} \,\chi_{c} - \rho_{\text{s}} \,\chi_{\text{s}} } \right)}}{{\,2\,\mu_{\text{0}} }}\,\frac{\partial }{\partial r}\left( {B^{\text{2}} } \right)\;} \\ 0 \\ { - \left( {\,\,\rho_{c} \, - \rho_{\text{s}} } \right)\,g\; + \;\frac{{\left( {\,\rho_{c} \,\chi_{c} - \rho_{\text{s}} \,\chi_{\text{s}} } \right)}}{{\,2\,\mu_{\text{0}} }}\,\frac{\partial }{\partial z}\left( {B^{\text{2}} } \right)\;} \\ \end{array} } \right)^{\text{T}} .$$

*ρ*_c_, *ρ*_s_, and *g*, are the crystal density (kg/m^3^), solution density (kg/m^3^), and gravitational acceleration (m/s^2^), respectively. *χ*_c_ and *χ*_s_ are the mass magnetic susceptibilities (m^3^/kg) of the HEWL crystals and the protein solution, respectively. In this study, *χ*_s_ is paramagnetic and *χ*_c_ is diamagnetic. Hence, the sign of the term of *ρ*_c_*χ*_c_ − *ρ*_s_*χ*_s_ becomes negative. *B*^2^ is calculated as *b*_*r*_^2^ + *b*_*z*_^2^ since *b*_*θ*_ becomes zero by the axisymmetry of ***B***. The sign of $\frac{\partial }{\partial r}\left( {B^{\text{2}} } \right)\;$ is positive where it is within the magnet coil radius. The sign of $\frac{\partial }{\partial z}\left( {B^{\text{2}} } \right)\;$ is negative where z > 0, and is positive where z < 0. In summary, the sign of *MF*_*r*_ is negative and that of *MF*_*z*_ depends on the location. Since the magnitude of *χ*_s_ is much higher than that of *χ*_c_, the repulsive force (d) plays a major role in moving the crystals. In addition, since *ρ*_c_ (1.24 × 10^–3^ kg/mL^24^) is only a bit larger than *ρ*_s_ (experimentally 1.09 × 10^–3^ kg/mL in this study), 87.9% of the crystals’ gravity is cancelled in the solution. That is, the magnitude of the term of *BF* is smaller than that of the gravity.

According to our experiment, the maximum magnitude in the term of $\frac{1}{\,2\,}\,\frac{\partial }{\partial z}\left( {B^{\text{2}} } \right)\;$ in Eq. (3) was 20.79 T^2^/m when *B* was applied 2.12 T at the origin O. In the same magnetic condition, the magnitude of $\frac{1}{\,2\,}\,\frac{\partial }{\partial r}\left( {B^{\text{2}} } \right)\;$ was 3.468 T^2^/m. In short, *MF*_*r*_ was as small as about 16.7% at its maximum compared to *MF*_*z*_. Even at such a weak force, *MF*_*r*_ can be tangible as a driving force on the convection and mass transfer phenomena^25,26^ in weightlessness. Furthermore, *MF*_*r*_ is free from the effect of gravity. These characteristics are very favorable for the control over the nuclei. In our experiment, regardless of *χ*_c_ and *χ*_s_ unknown, the contactless crystallization was realized without using these properties. This technical procedure is presented in the Appendix 2 attached as the Supplementary Material.

### Dynamical stability

In our search for creating contactless crystallization, we aimed to establish a stable condition during the crystal growth process. The schematic illustration in Fig. 1 represents the isothermal distribution of the magnitude of the magnetic force, which was obtained by the computational approach. This approach is described in Appendix 1. Points *A* to *I* in this figure are the representative points, and point *C* is the MMF point.

As a first thought experiment, we assumed the case in which the force balance on the crystals is weightless at point *C*. This case is presented in Fig. 1, where the blue isothermal distribution corresponds to a weak gravity environment and the red distribution corresponds to a hypergravity one. Notice that the strength of the magnetic force and the gravity in Fig. 1 are displayed as dimensionless. We can find an hourglass-shaped distribution near point *C*. This is attributed to the results that the *MF*_*z*_ cancels out the *BF* at the *C*, whereas the *MF*_*r*_ remains. The hourglass-shaped distribution of the magnetic force was already confirmed visually in a previous study^26^.

In the next thought experiment, the magnitude of *MF*_*z*_ is slightly enhanced. Since the magnitude of the upward magnetic force exceeds that of the gravity, the initial weightless condition at the *C* is changed to the other positions of *B* or *D*. In this thought, we assume the cases in which weightlessness is realized at the *B*. Therefore, the magnitudes of *MF*_*z*_ at the *A*, *B*, and *C* are arranged in the following relationship: $\left. {MF_{z} } \right|_{A} < \left. {MF_{z} } \right|_{B} < \left. {MF_{z} } \right|_{C}$. The force balance between *BF* and *MF*_*z*_ is then deduced as:

4 $$\left. {BF + MF_{z} } \right|_{A} < \left. {0,\quad BF + MF_{z} } \right|_{B} = 0,\quad \left. {BF + MF_{z} } \right|_{C} F > 0.$$

Relation (4) indicates a preferable condition, where a “*stable*” condition is established in respect to the vertical direction.

When we assume in a third thought experiment that the weightlessness is realized at the position of *D*, the force magnitude at the *C*, *D*, and *E* are presented as $\left. {MF_{z} } \right|_{C} > \left. {MF_{z} } \right|_{D} > \left. {MF_{z} } \right|_{E}$. Consequently, the following relationship is deduced as:

5 $$\left. {BF + MF_{z} } \right|_{C} > \left. {0,\quad BF + MF_{z} } \right|_{D} = 0,\quad \left. {BF + MF_{z} } \right|_{E} < 0.$$

Relation (5) represents that a slight unbalance in the force is positively increased there, and the HEWL crystals cannot stay levitated. That is, the force balance is “*unstable*”. This indicates that the vertically stable condition is limited to only a specific position in the magnet.

As regards the radial direction, the bore axis is always “*stable*” since the *MF*_*r*_ becomes zero. In particular, this effect is remarkable in the hourglass-shaped distribution in Fig. 1. For example, the crystals at the *I* are easily driven to the *D* according to the relationship on the radial magnetic force of $\left. {MF_{r} } \right|_{D} < \left. {MF_{r} } \right|_{I}$. On the other hand, it is impossible for the *D* to hold the crystals stably because this point is dynamically a “*saddle*” owing to the vertical force balance in Eq. (5). From the viewpoint of mechanical engineering, we will hereafter use the term “*dynamic* *stability*” on the contactless crystallization. In short, the crystallization should be carried out “*above* *the* *MMF* *point*” and “*on* *the* *bore* *axis*”. The key objective in our technique is how to locally strengthen the *MF*_*r*_ without disturbing the influence of *MF*_*z*_. These results are summarized in Table 1.

**Table 1:** The summary of the force balance between the buoyancy force (*BF*) and vertical component of the magnetic force (*MF*_*z*_). We assume the vertically upward force is positive and the point *C* is the MMF point. So the relationships of *BF* < 0, *MF*_*z*_ > 0, $\left. {MF_{z} } \right|_{A} < \left. {MF_{z} } \right|_{B} < \left. {MF_{z} } \right|_{C}$, and $\left. {MF_{z} } \right|_{E} < \left. {MF_{z} } \right|_{D} < \left. {MF_{z} } \right|_{C}$ are regarded.

Points in Fig. 1	When *BF* + *MF*_*z*_ is zero at the point *B*	When *BF* + *MF*_*z*_ is zero at the point *C*	When *BF* + *MF*_*z*_ is zero at the point *D*
A	$\left. {BF + MF_{z} } \right|_{A} \; < \;\;0$, crystals move downward	$\left. {BF + MF_{z} } \right|_{A} \; < \;\;0$, crystals move downward	
B	$\left. {BF + MF_{z} } \right|_{B} \; = \;\;0$, crystals are in the weightlessness, and they can keep stable levitation (dynamically stable)	$\left. {BF + MF_{z} } \right|_{B} \; < \;\;0$, crystals move downward	
C (MMF point)	$\left. {BF + MF_{z} } \right|_{C} \; > \;\;0$, crystals move upward	$\left. {BF + MF_{z} } \right|_{C} \; = \;\;0$, crystals are in the weightlessness, but they cannot keep stable levitation simply by the condition at C	$\left. {BF + MF_{z} } \right|_{C} \; > \;\;0$, crystals move upward
D		$\left. {BF + MF_{z} } \right|_{D} \; < \;\;0$, crystals move downward	$\left. {BF + MF_{z} } \right|_{D} \; = \;\;0$, crystals are in the weightlessness, but they cannot keep stable levitation (dynamically unstable)
E		$\left. {BF + MF_{z} } \right|_{E} \; < \;\;0$, crystals move downward	$\left. {BF + MF_{z} } \right|_{E} \; < \;\;0$, crystals move downward

## Methods and equipment

### Our designed magnetic force booster

**Figure 2:**
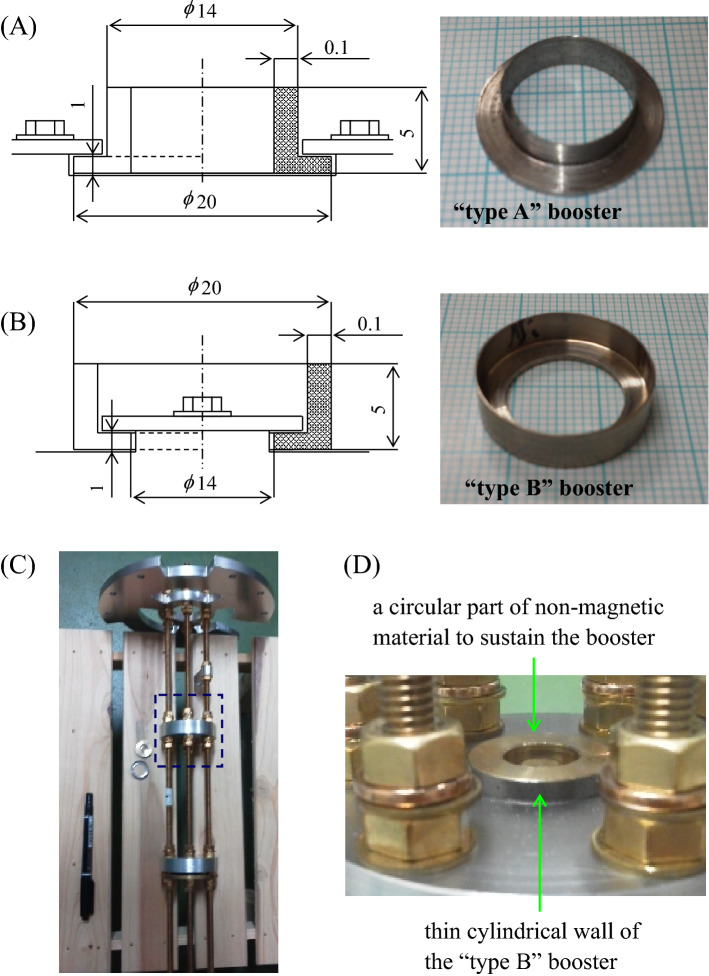
Schematic illustrations and actual photos of newly developed magnetic force boosters (2 types). The magnetic force booster of (**A**) type A, and (**B**) type B. Both boosters are made of stainless steel (ISO X6Cr17 (AISI-430 alloy). The volume of the type A booster is 182 mm^3^, and the type B booster is 185 mm^3^. A thin cylindrical wall (0.1 mm), common to both types, is essentially important to locally strengthen the *MF*_*r*_. (**C**) shows the overall photo including the booster and a nonmagnetic support table. The booster was set on the table where the broken line is presented. (**D**) Close-up photo of the type B booster arranged on the support table. This is fixed by sandwiching the bottom with a circular plate of non-magnetic material.

In relation to the previous section, the basic performance required for a new booster is to keep a dynamically stable condition during the crystal growth. Since few examinations of the magnetic force booster have been reported, we conducted many trial tests to find the most appropriate shape and material. All of the trial boosters were designed to be a much smaller size than that of the prototype booster^6^. In our test, we succeeded in realizing contactless crystallization with two types of boosters. Figure 2 shows the schematic illustrations and actual photos of our boosters. In this paper, Fig. 2A shall be named “type A” and Fig. 2B “type B”. Both boosters are made of stainless steel (ISO X6Cr17 (AISI-430 alloy)) and are 20 mm in diameter. The “type A” is fixed by holding the brimmed flange. The “type B” is fixed by sandwiching the inner bottom with a circular plate of non-magnetic material. A thin cylindrical wall (0.1 mm thickness), which both types have in common, is essentially important to locally strengthen the *MF*_*r*_. The results from using the type B booster are presented in detail in this paper. Figure 2C is the overall photo, including the booster and a nonmagnetic support table. The booster was put on the table, as shown by the broken line. Figure 2D is a close-up photo of the type B booster arranged on the table.

As presented in the drawings of Fig. 2, the volumes of the type A and B boosters are estimated as 182 and 185 mm^3^, respectively. The prototype booster developed at NIMS, in contrast, was made up of two parts; one was a disk-shaped object (22 mm diameter and 10 mm height), and the other was a cylindrical object (40 mm outer diameter, 20 mm inner diameter, and 10 mm height), and the volume was 13,226 mm^3^ in total. Our miniaturized boosters are devoted to simplifying the observation systems, and are therefore versatile for saving a lot of time and effort in exchanging samples. It can be safely sustained in a magnetic field even with small supports.

### Crystallization and the booster setting

The crystals were adjusted by the batch method. The final concentrations of the HEWL (MP Biomedicals, Inc.), hydrochloric acid (Wako Co. Ltd), and the gadolinium chloride hexahydrate (Wako Co. Ltd) were 6.44 wt%, 0.00535 mol/kg, and 0.388 mol/kg, respectively. We dissolved them in water in that order, then this protein solution was poured into a glass vessel and installed in a superconducting magnet (JMTD-6T100EF3, JASTEC), where the type B booster was installed in advance. The center part of the solution was accorded to a place about 3 mm above the MMF point. The booster’s upper edge was set at 25 mm below the MMF point. The magnetic flux density at the origin O (the center of the coil) was set at 2.12 T. This was equivalent to 1.75 T at the buster position, and we could find that the booster was magnetized. The temperature in the bore was kept constant at 17 ± 0.2 °C, and was measured at 2 min intervals by means of a data logger (NR-1000, Keyence Co. Ltd.) and several thermocouples (T type, Anbe SMT Co. Ltd.). The crystallization process was recorded from the side by using a rigid scope (Industrial Rigid Scope Type 5, Olympus Co. Ltd.) and a CCD camera (OH414, Olympus Co. Ltd.).

### Magnetic saturation of the booster

When using a magnetic force booster made of iron, we must consider the magnetic saturation of the booster. In our experiment, the booster was magnetically saturated, since a magnetic field of 2 T or more was applied. This suggests that the magnetic force by the booster had reached almost constant magnitude. We know the radial component of the magnetic force by the superconducting magnet becomes almost zero near the MMF point. This means that the driving force on the crystals can be controlled simply by the vertical component of the magnetic force by the superconducting magnet. That simple control over the crystals is very helpful for the reproducibility of the crystallization.

## Experimental results

**Figure 3:**
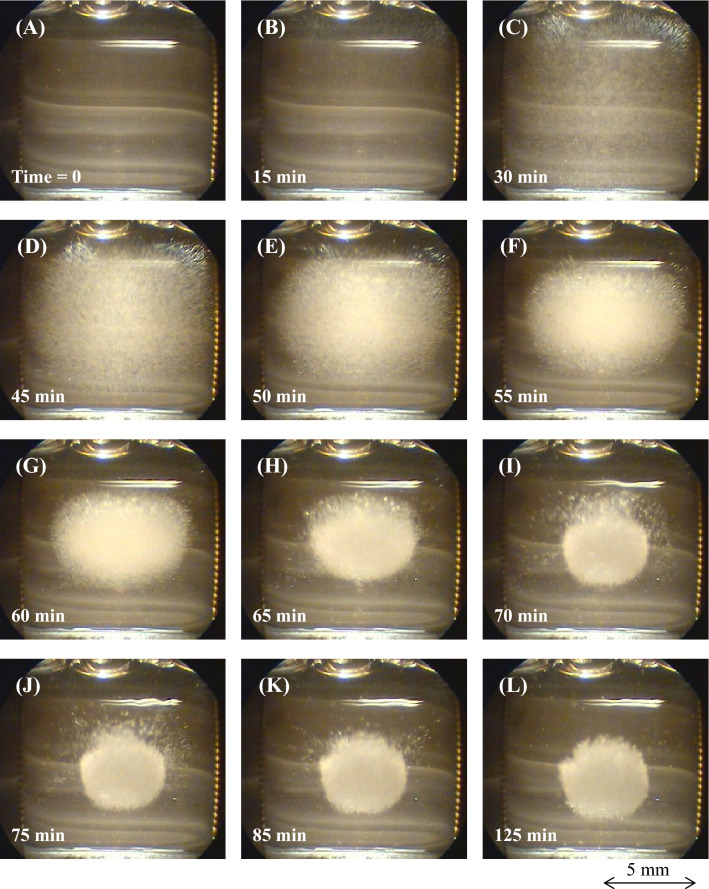
The series of photos of the contactless crystallization of HEWL. We used the type B booster, and the magnetic flux density at the coil center was 2.12 T. At 15 min, white fine crystallites appeared slightly above the solution. At 1 h, they slowly concentrated while levitating, and aggregated into a spherical shape. We kept the sphere crystal levitated all day and confirmed that it never contacted the vessel.

Figure 3 displays a series of photos in the crystallization process using the type B booster. Figure 3A shows the initial state. At 15 min, fine white crystallites appeared above the solution (Fig. 3B). After 30–45 min, they grew noticeably and could be found in the entire solution (Fig. 3C,D). As they slowly traveled to the vessel center (Fig. 3E), they aggregated together in a spherical shape (Fig. 3F–H) at 1 h. After 70 min, fine particles surrounding the spherical crystal gradually vanished (Fig. 3I–K). After about 2 h, the sphere crystal seen in Fig. 3L had grown to a larger size than that in Fig. 3I. We kept the crystal levitating all day, and we confirmed that it had never contacted the vessel. Thus, the sequence of photos in Fig. 3 provides evidence that we succeeded in the contactless crystallization of protein. As Supplementary Material in the crystallization process, a short movie recorded at 100 × speed (Supplementary Video 1) will be made available separately.

**Figure 4:**
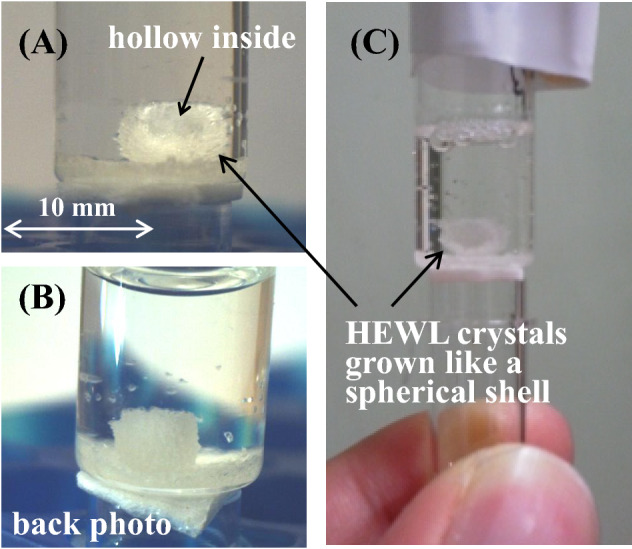
The spherical crystal photographed after the experiment. The crystal sphere was a polycrystal of HEWL. (**A**) Provides the evidence that the crystal was hollow inside (see the blue arrow). The black arrows in (**A**) and (**C**) point to where the crystals grew like a spherical shell.

Figure 4a–c are photos of the spherical crystal taken after the experiment. As pointed out by the arrows in Fig. 4A,C, its inside was hollow and the crystals (polycrystal) had grown into a spherical shell. As far as we know, such an unusual phenomenon has never been found before.

**Figure 5:**
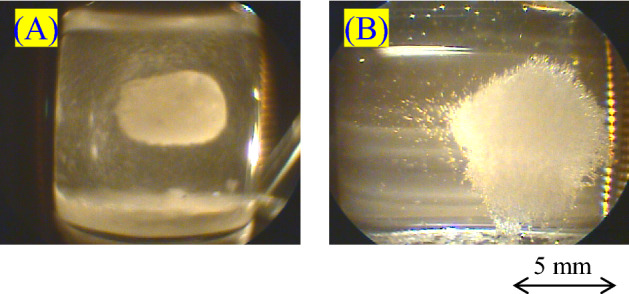
The contactless crystallization results verified the reproducibility. (**a**) Used the type A booster, and (**b**) used the type B booster. The spherical shell crystallization was reproduced only once, in (**a**). It is essentially important to configure strict and careful adjustments if we need to make the nuclei crystallize into a shell shape.

We verified the reproducibility of this method. As shown in Fig. 5, the contactless crystallization was performed more than five times, not only by the type B booster (Fig. 5b) but also by the type A booster (Fig. 5a). The spherical shell crystallization, however, could be reproduced only once, as seen in Fig. 5a. Without the booster, HEWL crystals never gathered in one point in the vessel. Those results confirm the significance of the magnetic force booster. We are convinced that the dynamically stable condition is essential for the contactless crystallization method.

## Discussion

**Figure 6:**
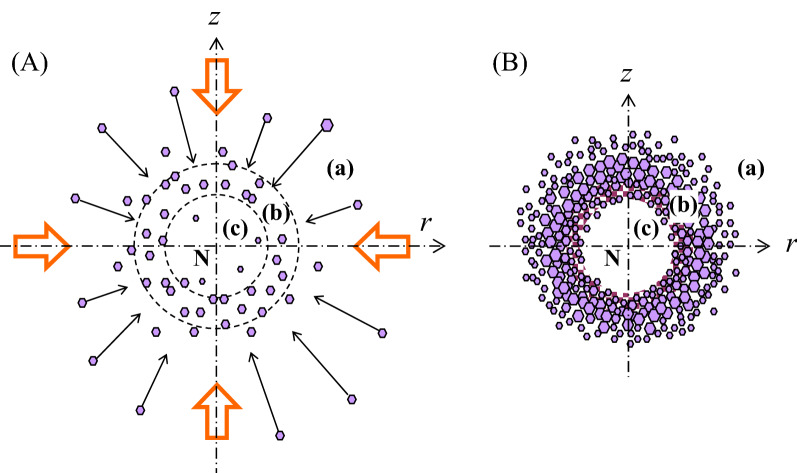
Schematic illustration of the spherical shell-shaped crystals. Point N is the center of the crystal. (**A**) Is the initial aggregation and (**B**) shows the well-grown state. When the nuclei generate in region (a), far away from N, they are strongly attracted, as shown by orange arrows. As the crystals get close to N, the external forces on the crystals become small, and finally the crystals are forced to stay near N. In the succession of other crystals’ concentrating, some crystals come into contact with the periphery crystals and grow large together. As a result, a spherical shell structure is shaped. Since the periphery part is constantly supplied with high-concentration protein molecules, the shell structure grows thick, as schematically shown in region (b). In contrast, mass transfer effect is suppressed in the hollow part of region (c). As a consequence, the crystals build a two-layer structure, i.e., a spherical shell outside and a hollow inside.

We demonstrated that the contactless crystallization is possible by using our magnetic force booster. We also found it essentially important to configure strict and careful adjustments if we need to make the nuclei crystallize into a shell shape. Here we will discuss the spherical shell-shaped structure by using the schematic illustrations in Fig. 6. As a prior assumption, the dynamical stability on the crystal growth must be stable, and the center of the spherical crystal is determined as the point N. As shown in Fig. 6, schema (A) and (B) indicate the initial aggregation and the well-grown state of the nuclei, respectively. When the nuclei generate in region (a) far away from the N, they are strongly attracted by the *RF*, as shown by the orange arrows in schema (A). Since the *MF*_*r*_ acts axisymmetrically and the vertical driving force *MF*_*z*_ + *BF* is also almost symmetric with respect to the N, fine crystals are driven to form in a quasi-spherical symmetry. A similar spherical polymer has been realized by the magnetic levitation technique^27^.

As the crystals get close to the N, the external forces on the crystals become smaller and smaller, and finally the crystals are forced to stay near the N. In the succession of the concentration of other crystals, some crystals come into contact with the periphery crystals and grow large together, resulting in the shaping of a spherical shell structure. Since the periphery part is constantly supplied with high-concentration protein molecules, the shell structure grows thick, as schematically shown in region (b). In contrast, the mass transfer effect is suppressed in the hollow part of region (c). The crystal growth becomes weak, and it is insufficient to fill up the hollow part with crystals. As a consequence, the crystals build a two-layer structure, i.e., a spherical shell outside and a hollow inside. If there were even a small disturbance in the crystals contacting each other, the spherical shell structure would probably be lost. In the file of Supplementary Material, Appendix 3 demonstrates the case in which the spherical crystal growth did not appear. In another case, Appendix 4 shows white precipitates aggregated spherically. Both crystallization conditions are the same as those in Fig. 3.

As regards the effect of gadolinium on the crystallization, it is unclear whether the presence of gadolinium atoms affects the crystal structure. We have confirmed that magnetic levitation is feasible even with nickel chloride and manganese chloride, and the use of a magnetic substance other than gadolinium has hardly any effect on changing the morphology of HEWL crystals. We are currently conducting research on the thermal properties of protein crystals^28^, and we suspect that magnetic substances might affect the thermophysical characteristics of the crystals. Further study is in progress.

In summary, our present opinion is that, even if a delicate experimental process is required, we would like to emphasize the merit of the magnetic force booster, which is the only way we know of to actually realize the contactless crystallization of protein. Further improvement of the booster and crystalline is in progress.

## Conclusions

In view of the dynamical stability of crystal nuclei, we developed a new type of magnetic force booster, and succeeded in crystallizing protein (hen egg white lysozyme) while making it levitate in a solution. The greatest novelty of our “*contactless* *crystallization*” method is that it holds great potential for the ability to control protein crystal growth from the early stages of nucleation. Our technique is crucially innovative in this point, especially in the field of protein crystal engineering. We hope that the method of using a magnetic force booster will be in widespread use in various fields such as materials engineering and applied chemistry.

## Supplementary Information

Supplementary Information 1.

Supplementary Information 2.

Supplementary Video 1.

## Data Availability

We wish to make our data (video movie and images) available to the public because they are very interesting and we think they are academically so important. In this paper, we attached a downsized 100 × speed movie of 3.66 MB (labeled as “Supplementary Video 1”), which is shown in Fig. 3. The raw movie has the volume of 26.5 GB in total. The movie recorded at 10 × speed is 830 MB, and that at 100 × speed is 83 MB. We have no objection to making these data available to the public. We have no objection to making the images presented in Figs. 3, 4 and 5 and images of Appendix 2 and 3 (they are separately attached as the Supplementary Material) available, too. If any readers need them, they can contact us by e-mail at makisyou@ifst.ous.ac.jp.
