# Supplementary material for: Contactless crystallization method of protein by a magnetic force booster
Source: Sci Rep. 2022 Oct 14;12:17287. doi: 10.1038/s41598-022-21727-x (PMC9568516; doi:10.1038/s41598-022-21727-x)
Supplement: Supplementary file 1 — Supplementary Information 1. [file 41598_2022_21727_MOESM1_ESM.doc]

**Supplementary Material**

**Appendix 1**

**Computational approach.** The schematic illustration of (A) in Appendix 1 represents a conventional superconducting magnet. In this study, the magnetic force was generated in the bore of the diameter 100 mm. The distribution of the magnetic force in the bore was calculated as follows. First, we approximated that the superconducting magnet coil was a multi-layer coil in which many circular coils were regularly aligned. A magnetic field around one circular coil was calculated by the Biot-Savart equation. All the magnetic fields created by the circular coil were superimposed on each other, and we approximated this superimposed result as a magnetic field of the magnet (see the results (B) in Appendix 1). The distribution of the magnetic force was derived from the computation results of the magnetic field (see the results (C) in Appendix 1). In the figures of (B) and (C), the strength of the magnetic force and the gravity are displayed as dimensionless.

**Appendix 2**

**Techniques for achieving contactless crystallization.** As a preliminary experiment, HEWL crystals were generated in advance. These crystals were positioned above the MMF point in the bore and we gradually increased the magnetic field. By the visualization system, we observed the crystal levitation and identified the magnetic flux density B the moment when the crystals had just levitated in the liquid. In the next step, we prepared the same crystallization solution one more time, and crystal growth was performed at the same position while applying a magnetic field of the same strength. By this method, contactless crystallization could be reproduced many times even if some properties of χ_c_, χ_s_, ρ_c_, and ρ_s_ were unknown. This method is very simple and is probably available for other proteins as well. The key point of this method is to prepare the same crystallization conditions. This can be realized more accurately when prepared at the molar concentration than when prepared at the volume molar concentration.

**Appendix 3**

**Contactless crystallization when the spherical crystal growth did not appear**. The pink circles in the images (A) to (D) represent that fine crystal particles were levitating in the vicinity of a large polycrystal. These particles gradually disappeared as the polycrystal grew large (see the images (E) to (I)). The crystallization conditions were the same as those in Fig. 3.

**Appendix 4**

**White precipitates aggregated spherically.** In the beginning, white precipitates drifted all over the solution, as shown in the image (A). After that, the precipitates slowly aggregated in the center of the vessel (images (B) to (D)), and changed to form a spherical shape (images (E) to (F)). The crystallization conditions were the same as those in Fig. 3.

*φ* 680mm

bore diameter

*φ* 100 mm

944 mm

475 mm

210 mm

1041 mm

**Appendix 1**

(C)

(A)

(B)


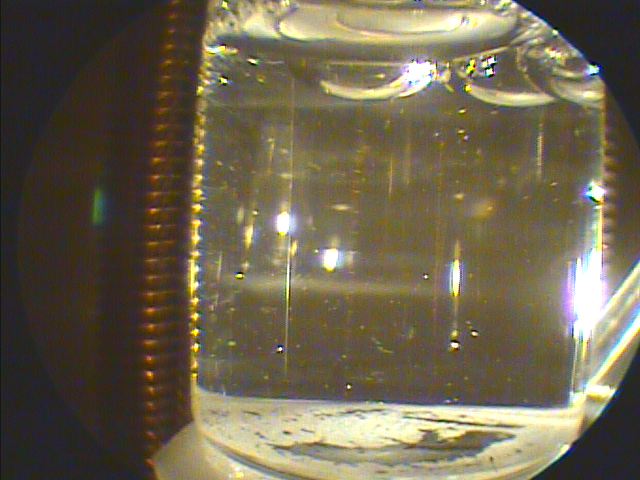

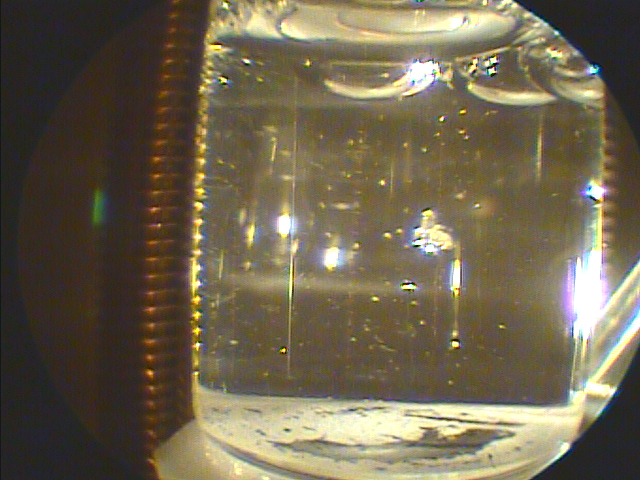

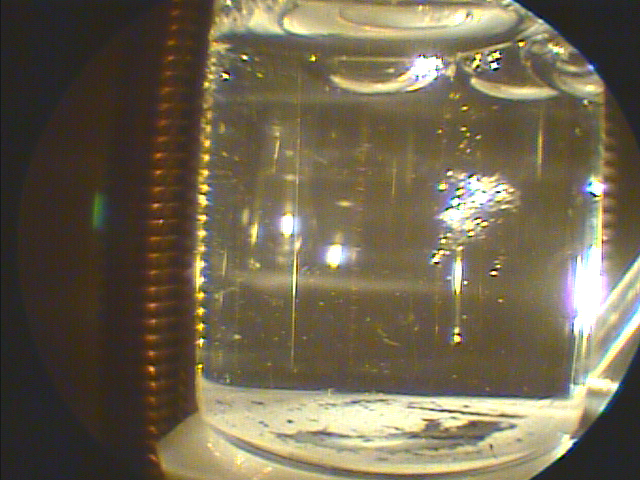


**(A)**

**(B)**

**(C)**


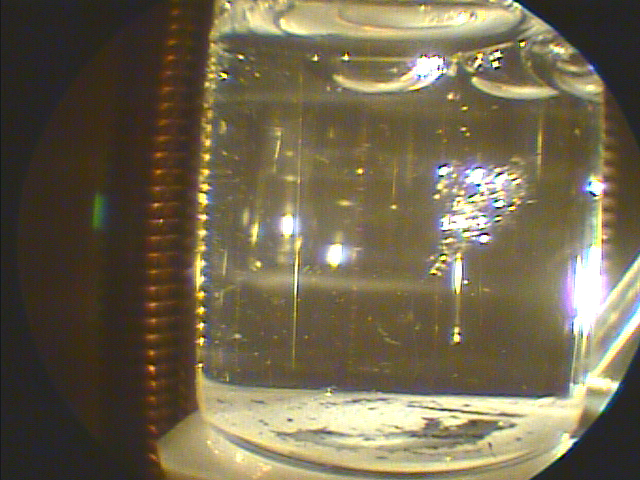

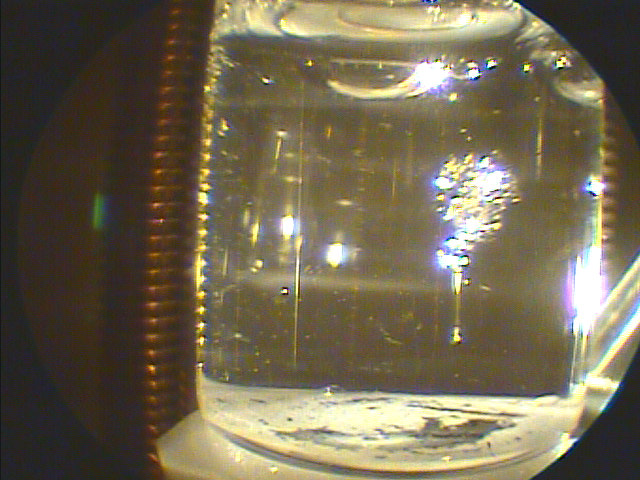

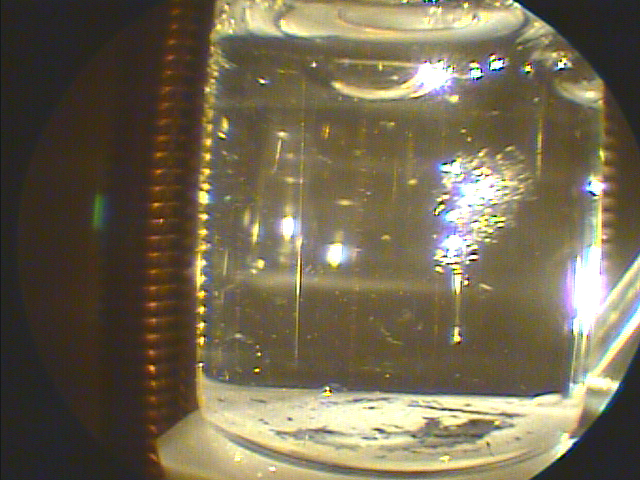


**(D)**

**(E)**

**(F)**

**4 h 50 min**

**1 h 40 min**

**3 h**


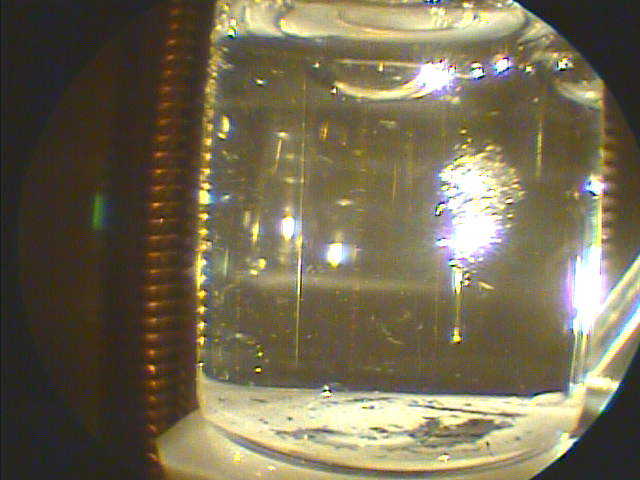

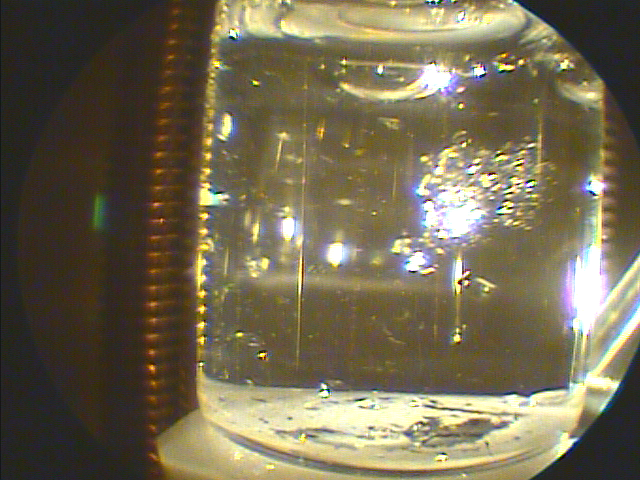

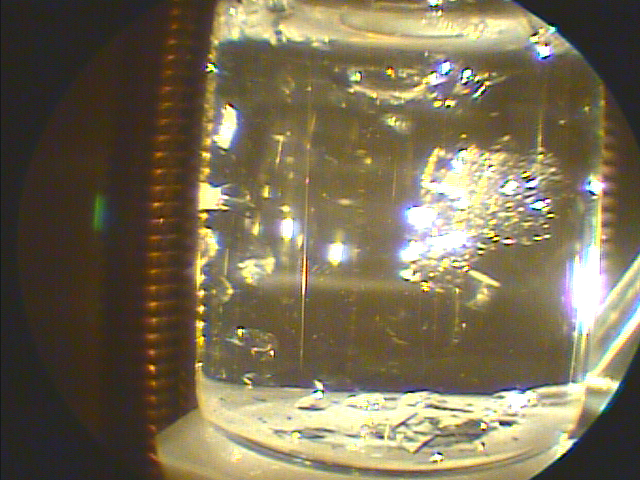


**(G)**

**(H)**

**(I)**

**11 h 10 min**

**14 h 50 min**

**24 h 30 min**

5 mm

**Appendix 3**

**6 h 20 min**

**7 h 50 min**

**9 h 40 min**


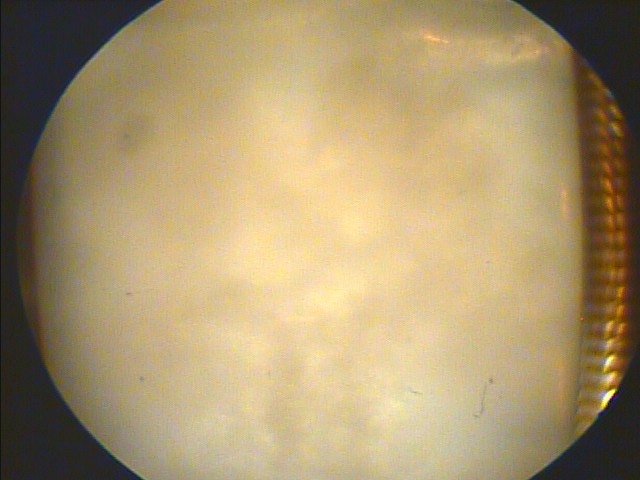

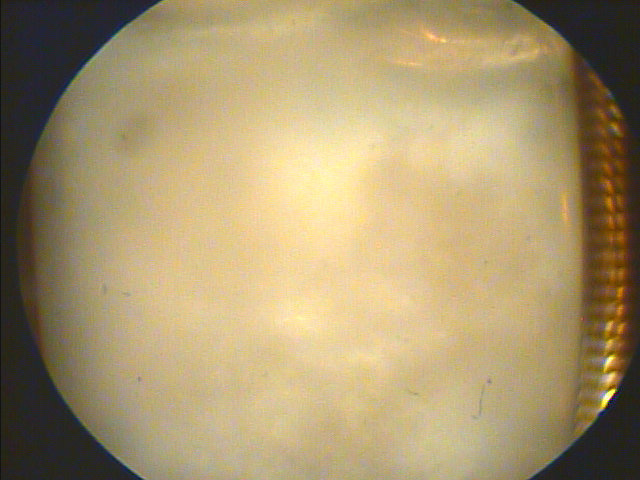

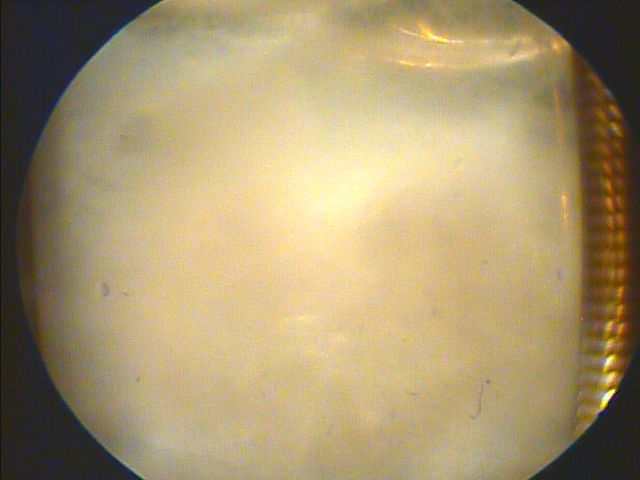

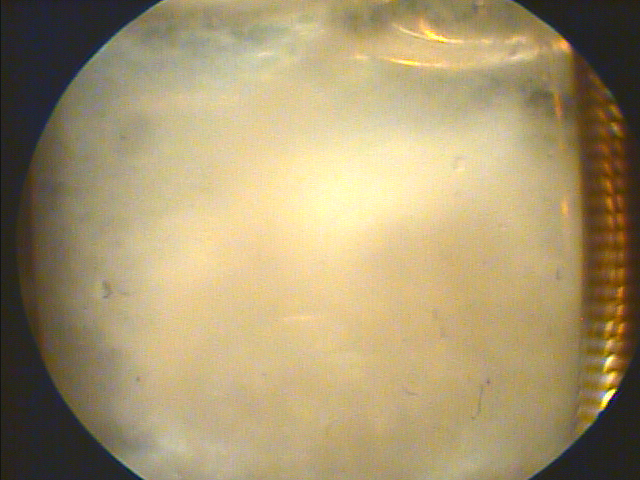

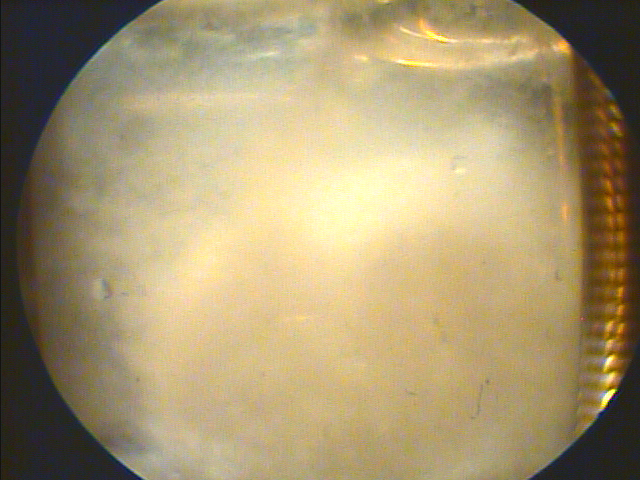

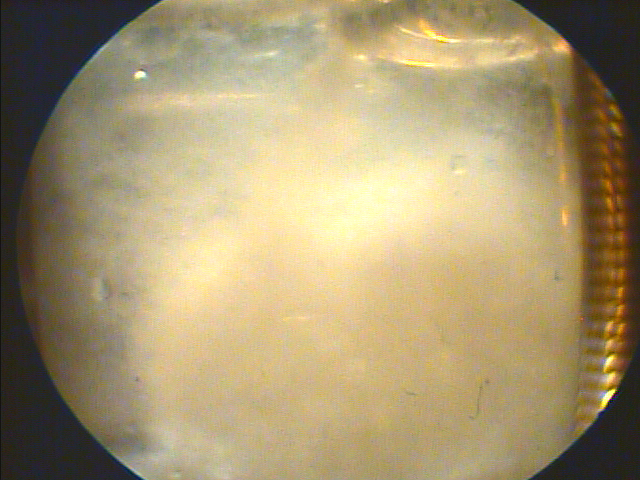


**(A)**

**(B)**

**(C)**

**(D)**

**(E)**

**(F)**

**6 h 40 min**

**7 h 40 min**

**8 h 30 min**

**1h 30 min**

**3 h 10 min**

**5 h 10 min**

5 mm

**Appendix 4**
