# Supplementary material for: Contactless crystallization method of protein by a magnetic force booster
Source: Sci Rep. 2022 Oct 14;12:17287. doi: 10.1038/s41598-022-21727-x (PMC9568516; doi:10.1038/s41598-022-21727-x)
Supplement: Supplementary file 2 — Supplementary Information 2. [file 41598_2022_21727_MOESM2_ESM.doc]

**Supplementary information**

**Movie legend for the “Supplementary Video 1”**

This short movie is a compressed version of the movie of crystal growth shown in Fig. 3, reproduced at 100x speed. The raw movie recording with standard speed has the volume of 26.5 GB in total. The movie recorded at 10x speed is 830 MB, and that at 100x speed is 83 MB. Since they are too large to show on the website of this journal, we will open a downsized 100x speed movie of 3.66MB, as a “Supplementary Video 1”.

The video is a little disturbed at the 37th second because the cassette tape was replaced, and the images during that time are cut off. That time is 20 seconds or less.

<Movie Contents>

This short movie observes the crystallization process using the magnetic force booster of “type B”. In the first stage of this movie, fine white crystallites appeared above the solution. After that, they grew noticeably and can be found in the entire solution. As slowly traveled to the vessel center, and they aggregated together in a spherical shape at 1 hour. After about 2 hours, the sphere crystal had grown to a larger size. From these results, our short movie provides good evidence that we succeeded in the contactless crystallization of protein by using our magnetic force booster. (We kept the crystal levitating all day, and we confirmed that it had never contacted the vessel. This is not included in this video)

In the article, we present in Fig. 4 that the inside of this spherical crystal was hollow and the crystals (polycrystal) had grown into a spherical shell. As far as we know, such an unusual phenomenon has never been found before. We discuss in the text how this “spherical shell-shaped structure” is realized. In the section of “Discussion”, we describe the crystallization process by using the schematic illustrations in Fig. 6.

Thank you very much for your interest in this video and our research.

*:._.:*~*:._.:*~*:._.:*~*:._.:*~*:._.:*~*:._.:*

Syou MAKI (Ph. D)

Associate Professor,

Institute of Frontier Science and Technology,

Okayama University of Science

1-1 Ridai-cho, Kitaku, Okayama, 700-0005 Japan

+81-86-256-9536

E-mail: [makisyou@ifst.ous.ac.jp](mailto:makisyou@ifst.ous.ac.jp)

*:._.:*~*:._.:*~*:._.:*~*:._.:*~*:._.:*~*:._.:*
